# Quality indicators for patients with traumatic brain injury in European intensive care units: a CENTER-TBI study

**DOI:** 10.1186/s13054-020-2791-0

**Published:** 2020-03-04

**Authors:** Jilske A. Huijben, Eveline J. A. Wiegers, Ari Ercole, Nicolette F. de Keizer, Andrew I. R. Maas, Ewout W. Steyerberg, Giuseppe Citerio, Lindsay Wilson, Suzanne Polinder, Daan Nieboer, David Menon, Hester F. Lingsma, Mathieu van der Jagt, the CENTER-TBI investigators and participants for the ICU stratum, Cecilia Åkerlund, Krisztina Amrein, Nada Andelic, Lasse Andreassen, Gérard Audibert, Philippe Azouvi, Maria Luisa Azzolini, Ronald Bartels, Ronny Beer, Bo-Michael Bellander, Habib Benali, Maurizio Berardino, Luigi Beretta, Erta Beqiri, Morten Blaabjerg, Stine Borgen Lund, Camilla Brorsson, Andras Buki, Manuel Cabeleira, Alessio Caccioppola, Emiliana Calappi, Maria Rosa Calvi, Peter Cameron, Guillermo Carbayo Lozano, Marco Carbonara, Ana M. Castaño-León, Simona Cavallo, Giorgio Chevallard, Arturo Chieregato, Mark Coburn, Jonathan Coles, Jamie D. Cooper, Marta Correia, Endre Czeiter, Marek Czosnyka, Claire Dahyot-Fizelier, Paul Dark, Véronique De Keyser, Vincent Degos, Francesco Della Corte, Hugo den Boogert, Bart Depreitere, Dula Dilvesi, Abhishek Dixit, Jens Dreier, Guy-Loup Dulière, Erzsébet Ezer, Martin Fabricius, Kelly Foks, Shirin Frisvold, Alex Furmanov, Damien Galanaud, Dashiell Gantner, Alexandre Ghuysen, Lelde Giga, Jagos Golubovic, Pedro A. Gomez, Francesca Grossi, Deepak Gupta, Iain Haitsma, Raimund Helbok, Eirik Helseth, Peter J. Hutchinson, Stefan Jankowski, Faye Johnson, Mladen Karan, Angelos G. Kolias, Daniel Kondziella, Evgenios Koraropoulos, Lars-Owe Koskinen, Noémi Kovács, Ana Kowark, Alfonso Lagares, Steven Laureys, Fiona Lecky, Didier Ledoux, Aurelie Lejeune, Roger Lightfoot, Alex Manara, Costanza Martino, Hugues Maréchal, Julia Mattern, Catherine McMahon, Tomas Menovsky, Benoit Misset, Visakh Muraleedharan, Lynnette Murray, Ancuta Negru, David Nelson, Virginia Newcombe, József Nyirádi, Fabrizio Ortolano, Jean-François Payen, Vincent Perlbarg, Paolo Persona, Wilco Peul, Anna Piippo-Karjalainen, Horia Ples, Inigo Pomposo, Jussi P. Posti, Louis Puybasset, Andreea Radoi, Arminas Ragauskas, Rahul Raj, Jonathan Rhodes, Sophie Richter, Saulius Rocka, Cecilie Roe, Olav Roise, Jeffrey V. Rosenfeld, Christina Rosenlund, Guy Rosenthal, Rolf Rossaint, Sandra Rossi, Juan Sahuquillo, Oddrun Sandrød, Oliver Sakowitz, Renan Sanchez-Porras, Kari Schirmer-Mikalsen, Rico Frederik Schou, Peter Smielewski, Abayomi Sorinola, Emmanuel Stamatakis, Nino Stocchetti, Nina Sundström, Riikka Takala, Viktória Tamás, Tomas Tamosuitis, Olli Tenovuo, Matt Thomas, Dick Tibboel, Christos Tolias, Tony Trapani, Cristina Maria Tudora, Peter Vajkoczy, Shirley Vallance, Egils Valeinis, Zoltán Vámos, Gregory Van der Steen, Jeroen T. J. M. van Dijck, Thomas A. van Essen, Roel P. J. van Wijk, Alessia Vargiolu, Emmanuel Vega, Anne Vik, Rimantas Vilcinis, Victor Volovici, Daphne Voormolen, Petar Vulekovic, Guy Williams, Stefan Winzeck, Stefan Wolf, Alexander Younsi, Frederick A. Zeiler, Agate Ziverte, Tommaso Zoerle, Hans Clusmann

**Affiliations:** 1 Department of Public Health, Center for Medical Decision Sciences, Erasmus MC– University Medical Center Rotterdam, Rotterdam, The Netherlands; 2 Division of Anaesthesia, University of Cambridge, Addenbrooke’s Hospital, Cambridge, UK; 3 Department of Medical Informatics, Amsterdam Public Health Research Institute, Academic Medical Center, University of Amsterdam, Amsterdam, The Netherlands; 4 Department of Neurosurgery, Antwerp University Hospital, University of Antwerp, Edegem, Belgium; 5 Department of Biomedical Data Sciences, Leiden University Medical Center, Leiden, The Netherlands; 6 School of Medicine and Surgery, University of Milan-Bicocca, Milan, Italy; 7 Neurointensive care, San Gerardo Hospital, ASST-Monza, Monza, Italy; 8 Division of Psychology, University of Stirling, Stirling, UK; 9 Department of Intensive Care Adults, Erasmus MC– University Medical Center Rotterdam, Rotterdam, The Netherlands

**Keywords:** Quality indicators, Benchmarking, Traumatic brain injuries, Intensive care units, Quality of health care

## Abstract

**Background:**

The aim of this study is to validate a previously published consensus-based quality indicator set for the management of patients with traumatic brain injury (TBI) at intensive care units (ICUs) in Europe and to study its potential for quality measurement and improvement.

**Methods:**

Our analysis was based on 2006 adult patients admitted to 54 ICUs between 2014 and 2018, enrolled in the CENTER-TBI study. Indicator scores were calculated as percentage adherence for structure and process indicators and as event rates or median scores for outcome indicators. Feasibility was quantified by the completeness of the variables. Discriminability was determined by the between-centre variation, estimated with a random effect regression model adjusted for case-mix severity and quantified by the median odds ratio (MOR). Statistical uncertainty of outcome indicators was determined by the median number of events per centre, using a cut-off of 10.

**Results:**

A total of 26/42 indicators could be calculated from the CENTER-TBI database. Most quality indicators proved feasible to obtain with more than 70% completeness. Sub-optimal adherence was found for most quality indicators, ranging from 26 to 93% and 20 to 99% for structure and process indicators. Significant (*p* < 0.001) between-centre variation was found in seven process and five outcome indicators with MORs ranging from 1.51 to 4.14. Statistical uncertainty of outcome indicators was generally high; five out of seven had less than 10 events per centre.

**Conclusions:**

Overall, nine structures, five processes, but none of the outcome indicators showed potential for quality improvement purposes for TBI patients in the ICU. Future research should focus on implementation efforts and continuous reevaluation of quality indicators.

**Trial registration:**

The core study was registered with ClinicalTrials.gov, number NCT02210221, registered on August 06, 2014, with Resource Identification Portal (RRID: SCR_015582).

## Background

Limited evidence is available to direct critical care practice in patients with traumatic brain injury (TBI) [1]. Randomized controlled trials have shown a limited potential to add evidence translatable to clinical practice, and new approaches are being explored to improve care, such as quality of care monitoring. Quality of care registration in patients with TBI could become part of an emerging international intensive care unit (ICU) or trauma registries [2–5]. When used over time and across centres, large datasets provide a rich source for benchmarking and quality improvement, i.e. with feedback on performance, between-centre discussions on policies, and opportunities to study best practice.

International registries can contribute to improved patient outcome, by identifying areas in need of quality improvement, informing health policies, and increasing transparency and accountability, as shown in other medical fields, like cancer [6], acute coronary syndrome [7], and cystic fibrosis [8]. Benchmarking TBI management between ICUs can only be reliable when standardized quality indicators are used and case-mix correction is applied [5]. Quality indicators can be subdivided into structure, process, and outcome indicators [9]. As no quality indicator set is available for patients with TBI, we recently performed a Delphi study to reach consensus on a quality indicator set [10].

The aim of the current study is to validate the consensus-based quality indicator set. We hereto analyzed patients enrolled in a large dataset of patients with TBI from the Collaborative European NeuroTrauma Effectiveness Research in Traumatic Brain Injury (CENTER-TBI) study. Data collected for CENTER-TBI included a comprehensive description of ICU facilities and patient outcomes in 54 centres, thus providing an opportunity to examine the usefulness of the newly developed indicator set [11]. Based on the validation result, the indicator set could be reduced to those that have the greatest potential for implementation.

## Methods

### Quality indicator set

In this validation study, we applied a previously developed quality indicator set based on a Delphi study to the CENTER-TBI study. The quality indicator set consisted of 17 structure, 16 process, and 9 outcome indicators for adult patients with TBI at the ICU. It was acknowledged that this initial set would be in need of further validation [10].

### Data

The CENTER-TBI study is a multicentre observational cohort study conducted in Europe, which recruited patients between 2014 and 2018 (Clinicaltrials.gov NCT02210221) [11, 12]. The core study contains 4509 patients. Inclusion criteria for the CENTER-TBI study were a clinical diagnosis of TBI, presentation within 24 h of injury, an indication for CT scanning, and the exclusion criterion was a pre-existing (severe) neurological disorder that could confound outcome assessments. We selected ICU patients for this study as the consensus-based indicators were specifically developed for the ICU. So, the inclusion criteria for our study were (1) admitted to the ICU and (2) adults older than 18 years. Processes of ICU care (vitals, treatments, and therapy intensity levels) were obtained on a daily basis. Outcomes were assessed at the ICU and at 3, 6, 12, and 24 months. In addition, questionnaires were completed by participating centres on structures and processes of care (Provider Profiling questionnaires [13]).

### Indicator scores

We determined whether the indicators could be calculated from the CENTER-TBI database and whether data collection fitted routine practice.

Structure indicator scores at centre level were calculated based on the Provider Profiling questionnaires and expressed as the number of centres that indicated that the structure was either present or absent.

Process indicators were calculated as the number of patients adherent to the indicator (numerator) divided by the number of patients to which the indicator could have applied per centre (denominator). The denominator could be based on a subset of patients (e.g. excluding patients with leg fractures for the indicator mechanical DVT prophylaxis).

(Crude) outcome indicators were calculated as the event rate of the indicator per centre (numerator) divided by the total number of patients which could have scored on the indicator (denominator). For the Glasgow Outcome Scale Extended (GOSE) and Short Form-36 version 2 (SF-36), the median scores were calculated.

Missing data were disregarded for the denominator so that the indicator adherence scores were based on the number of patients that could be exposed to the indicator. We present the median indicator numbers across centres with interquartile range.

### Validation of the quality indicators

The usefulness of the quality indicators was based on three criteria [14]: feasibility [15], discriminability [16, 17], and statistical uncertainty [15, 18, 19]. As no previous studies report thresholds on these criteria, we set a priori thresholds based on consensus.

#### Feasibility

Feasibility addresses data quality and ease of quality indicator calculation [15].

The feasibility was quantified by the completeness of the variables required to calculate the indicators. We set an arbitrary threshold of > 70% completeness of data (of denominator) to determine feasibility.

#### Discriminability

To determine discriminability (between-centre variation), we determined the between-centre differences in adherence to quality indicators to evaluate their potential for benchmarking and quality improvement [16, 17].

Between-centre variation for structure indicators was determined by the number of centres having that structure. We set an arbitrary threshold for moderate discriminability at 80–90% and for poor discriminability at 90–100% adherence to structure and process indicators. Such high levels of adherence decrease discrimination between centres.

The between-centre variation of process and outcome indicator scores, adjusted for case-mix and statistical uncertainty, was quantified with the median odds ratio (MOR) [20]. The MOR represents the odds of being adherent to a specific indicator for two patients with the same patient characteristics from two randomly selected centres. The higher the MOR, the larger the between-centre variation (a MOR equal to 1 reflects no variation).

For process and outcome indicators, we considered a low (unadjusted) interquartile range on scores (IQR < 10) or non-significant (adjusted) between-centre differences or a MOR of 1.1 or less as poor discriminability. Case-mix- and uncertainty-adjusted process and outcome indicator scores per centre were presented in caterpillar plots.

#### Statistical uncertainty

Reliability refers to the reproducibility of a quality indicator and is threatened by unclear indicator definitions [15] and statistical uncertainty [18, 19]. We determined whether we could calculate indicators in a uniform way or made minor changes to definitions. Statistical uncertainty was determined by random variation due to low numbers of events (only applicable to outcome indicators).

Statistical uncertainty for outcome indicators was determined by the median number of events across centres. We set the threshold for high statistical uncertainty at < 10 events.

### Statistical analysis

Baseline centre and patient characteristics are described as frequencies and percentages. Between-centre variation of process and outcome indicator scores was calculated with a random-effect logistic regression analysis. We used a random effect model (random effect for centre) to account for the fact that indicator scores in centres with a small number of patients can have extreme values due to random variation. Also, only centres with > 10 admitted ICU patients were included. To correct for case-mix, we used the extended International Mission for Prognosis and analysis of Clinical Trials in TBI (IMPACT) prognostic model: core (age, motor score, pupillary light reactivity), CT (hypoxia, hypotension, epidural hematoma, traumatic subarachnoid hemorrhage, and Marshall CT classification) and lab (first glucose and hemoglobin) [21], and injury severity score (ISS). The MOR was calculated from the *τ*^2^ (variance of random effects).

Case-mix- and uncertainty-adjusted process and outcome indicator scores per centre are presented in ‘caterpillar’ plots. *p* values for determining the significance of the between-centre variation were calculated with a likelihood ratio test comparing a model with and without a random effect for centre. A mixture distribution is required to calculate the *p* value as the null hypothesis is on the boundary of the parameter space [22].

For the calculation of random effect models, missing data were imputed with multiple (*N* = 5) imputation with the MICE package from R [23]. Statistical analyses were performed in R statistical software. Neurobot version 2.1 (data extraction date 23-12-2019) was used.

## Results

A total of 26 (11 structure, 8 process, and 7 outcome indicators) of the 42 indicators of the Delphi set could be extracted from the CENTER-TBI database. (Additional file 1).

### Baseline data

Fifty-four centres from 18 countries were included, totaling 2006 adult patients. The median number of ICU patients included per centre was 23 (IQR12–43, range 2–119). Centres were mostly academic centres (*N* = 51; 94%) and designated as level I trauma centres (*N* = 37; 69%). Most centres were located in Northern (*N* = 20; 37%) or Western Europe (*N* = 19; 35%) (Table 1).

**Table 1:** Baseline centre and patient characteristics

Centre characteristics	Centre level (*N* = 54)			Patient level (*N* = 2006)	
	*N*	%		*N*	%
Centre					
Academic	51/54	94		1901/2006	95
Nonacademic	3/54	6		105/2006	5
Centre location^a^					
Urban	53/54	98		1990/2006	99
Suburban	1/54	2		16/2006	1
Trauma designation^b^					
Level I	37/54	69		1468/2006	73
Level II	4/54	7		84/2006	4
Level III	1/54	2		135/2006	7
No designation/NA	12/54	22		319/2006	16
Electronic patient records at the ICU					
Yes	42/54	78		1690/2006	84
No	12/54	22		316/2006	16
Location^c^					
Northern Europe	20/54	37		650/2006	33
Western Europe	19/54	35		809/2006	40
Southern Europe	12/54	22		524/2006	26
Eastern Europe	2/54	4		22/2006	1
Israel	1/54	2		1/2006	0
Patient characteristics	Centre level (*N* = 54)			Patient level (*N* = 2006)	
	Median %	IQR	Min-max	*N*	%
Age (years)^d^					
Adults (≥ 18 < 65 years)	74	63–84	0–100	1454/2006	72
Elderly (≥ 65 years)	26	16–37	0–100	552/2006	28
Gender					
Male	76	67–83	55–100	1479/2006	74
Female	25	19–33	6–46	527/2006	26
TBI severity (GCS)^e^					
Mild 13–15	34	22–43	5–100	671/1891	35
Moderate 9–12	17	11–21	4–38	305/1891	16
Severe 3–8	53	40–61	18–100	915/1891	48
ISS score					
< 16	7	3–14	1–24	76/1963	4
≥ 16	100	96–100	76–100	1887/1963	96
AIS^f^					
Thorax/chest ≥ 3	33	20–40	8–100	654/2006	33
Abdomen/pelvis ≥ 3	9	6–13	1–33	173/2006	9
Cause of injury					
Road traffic incident	45	35–55	0–68	849/1921	44
Incidental fall	40	33–50	11–100	802/1921	42
Violence/assault	2	0–7	0–43	83/1921	5
Suicide attempt	0	0–3	0–20	44/1921	2
Other	6	0–11	0–38	143/1921	7

This table describes the centre characteristics (at centre level) and the entire ICU population (patient level)

*AIS* Abbreviated Injury Scale, *GCS* Glasgow Coma Scale, *ICU* intensive care unit, *ISS* injury severity scale, *NA* not applicable, *TBI* traumatic brain injury

^a^Urban: A hospital location very near to a city and situated in a crowded area. Suburban: between urban and rural (an hospital location in or very near to the countryside in an area that is not crowded)

^b^Location is based on United Nations geoscheme: Northern Europe = Norway (*N* = 163), Sweden (*N* = 87), Finland (*N* = 132), Denmark (*N* = 3), the UK and Ireland (*N* = 271), and Baltic States: Latvia (*N* = 10), Lithuana (*N* = 23); Western Europe = Austria (*N* = 109), Belgium (*N* = 193), France (*N* = 115), Germany (*N* = 87), and the Netherlands (*N* = 359); Southern Europe = Serbia (*N* = 10), Italy (*N* = 293), and Spain (*N* = 195); Eastern Europe = Romania (*N* = 3), Hungary (*N* = 20);

^c^Level I trauma centre: A regional resource centre that generally serves large cities or population-dense areas. A level I trauma centre is expected to manage large numbers of severely injured patients (at least 1200 trauma patients annually or have 240 admissions with an injury severity score of more than 14). It is characterized by a 24-h in-house availability of an attending surgeon and the prompt availability of other specialties (e.g. neurosurgeon, trauma surgeon). Level II trauma centre: A level II trauma centre provides comprehensive trauma care in either a population-dense area in which a level II trauma centre may supplement the clinical activity and expertise of a level I institution or occur in less population-dense areas. In the latter case, the level II trauma centre serves as the lead trauma facility for a geographic area when a level I institution is not geographically close enough to do so. It is characterized by a 24-h in-house availability of an attending surgeon and the prompt availability of other specialties (e.g. neurosurgeon, trauma surgeon). Level III trauma centre: A level III trauma centre has the capacity to initially manage the majority of injured patients and have transfer agreements with a level I or II trauma centre for seriously injured patients whose needs exceed the facility’s resources

^d^The number of centres that admitted children was 27; therefore, the distribution and median is skewed towards 1%. One centre included 1 patient that was an elderly person (therefore max = 100%)

^e^GCS at baseline: Post stabilization value, if absent prehospital values are used. Intubated/untestable verbal (*V*) scores are treated as unknown

^f^AIS score of 3 or more reflects serious extracranial injury

Around 28% of patients admitted to ICU were older than 65 years and mostly male (*N* = 1561; 73%). According to the baseline GCS score, 48% had severe (GCS < 9; *N* = 915), 16% moderate (GCS 9–12; *N* = 305), and 48% mild TBI (GCS 13–15; *N* = 671). The majority of patients (*N =* 1963; 96%) suffered from polytrauma. The cause of injury was mostly related to road traffic accidents (*N* = 849; 44%) or incidental falls (*N* = 802; 42%) (Table 1).

### Adherence

Regarding structure indicators, sub-optimal adherence rates were found for most indicators, including the presence of a neuro-ICU (*N* = 35; 65%), operation room availability 24 h per day (*N* = 40; 75%), and presence of a step-down unit (*N* = 38; 70%) (Additional file 2). Patient-to-nurse ratio’s varied, with reported ratios of 1 (*N* = 14; 26%), 1–2 (*N* = 23; 43%), and 2–3 (*N* = 17; 31%) patients per nurse. Adherence was high for ‘the existence of a protocol including specific guidelines’ (*N* = 47; 89%), ‘protocol for glucose management’ (*N* = 43; 81%), ‘the availability of a neurosurgeon within 30 minutes after call’ (*N* = 49; 93%), and ‘the 24/7 availability of a CT scan and radiologist review’ (*N* = 50; 91%).

Sub-optimal adherence rates were found for most process indicators, including ICP monitoring in the severe TBI group (median 69%, IQR 44–82), basal caloric intake within 5–7 days (*N* = 20%, IQR 3–47), and ‘patients that receive DVT prophylaxis with low molecular weight heparins’ (median 63%, IQR 49–78) (Additional file 3). Adherence was high for ‘enteral nutrition within 72 hours’ (median 99%, IQR 87–100).

For outcome, the centres had a median [IQR] ICU mortality of 12% [9–21], ventilator-acquired pneumonia (VAP) incidence of 14% [0–31], and hyperglycemia incidence of 35% [22–45]. The median [IQR] GOSE was 5 [3–7], the SF-36v2 physical component summary (PCS) 46 [37–54], and SF-36v2 mental component summary (MCS) was 46 [36–55] (Additional file 4).

### Feasibility

Feasibility of structure indicators was generally high (overall more than 98% available data). Feasibility was low for one process indicator: ‘mechanical DVT prophylaxis within 24 hours’ (43% available data). Feasibility was high for outcome indicators, except for the SF-36 MCS and PCS scores (28% available data) collected after 6 months (due to loss to follow-up) (Additional files 2, 3, 4).

Overall, one process and one outcome indicator showed low feasibility (Table 2).

**Table 2:**
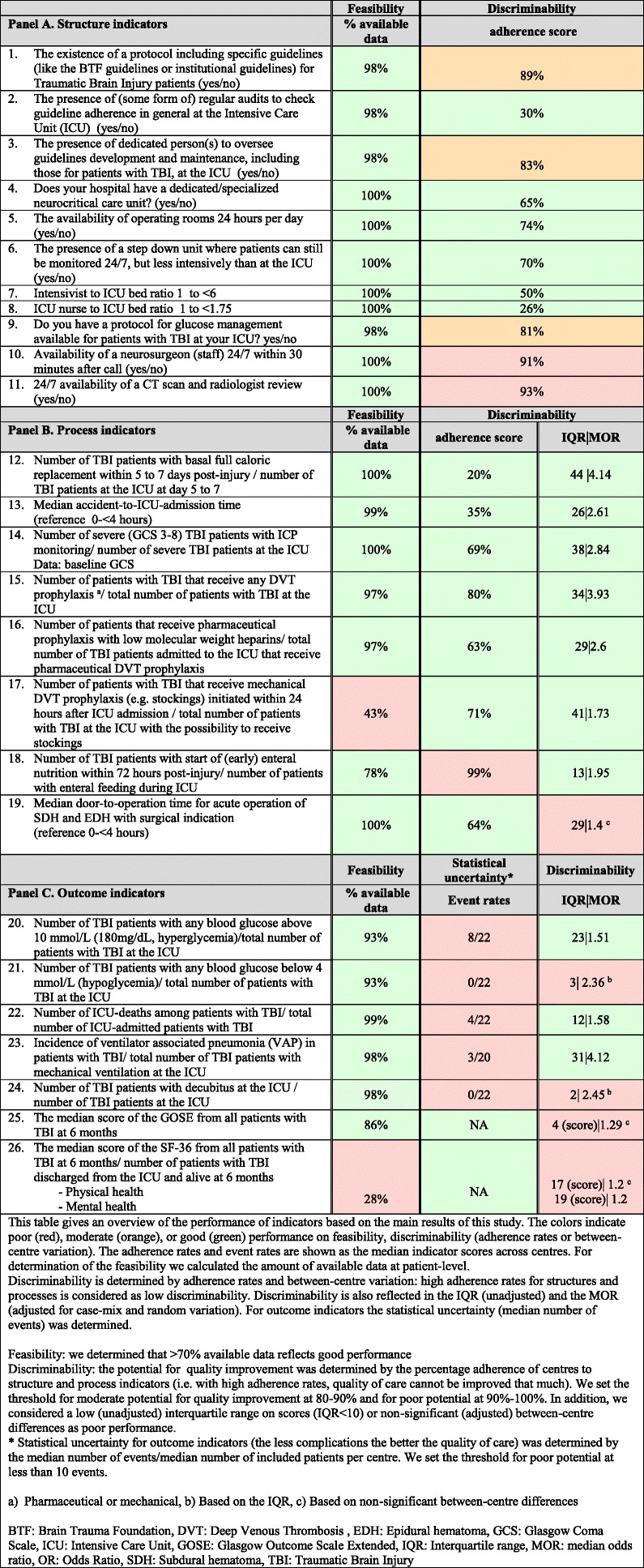
Overview of indicator performance

This table gives an overview of the performance of indicators based on the main results of this study. The colors indicate poor (red), moderate (orange), or good (green) performance on feasibility and discriminability (adherence rates or between-centre variation). The adherence rates and event rates are shown as the median indicator scores across centres. For the determination of the feasibility, we calculated the amount of available data at patient level

Discriminability is determined by adherence rates and between-centre variation: high adherence rates for structures and processes are considered as low discriminability. Discriminability is also reflected in the IQR (unadjusted) and the MOR (adjusted for case-mix and random variation). For outcome indicators, the statistical uncertainty (median number of events) was determined

Feasibility: we determined that > 70% available data reflects good performance

Discriminability: the potential for quality improvement was determined by the percentage adherence of centres to structure and process indicators (i.e. with high adherence rates, quality of care cannot be improved that much). We set the threshold for moderate potential for quality improvement at 80–90% and for poor potential at 90–100%. In addition, we considered a low (unadjusted) interquartile range on scores (IQR < 10) or non-significant (adjusted) between-centre differences as poor performance

*BTF* Brain Trauma Foundation, *DVT* deep venous thrombosis, *EDH* epidural hematoma, *GCS* Glasgow Coma Scale, *ICU* intensive care unit, *GOSE* Glasgow Outcome Scale Extended, *IQR* interquartile range, *MOR* median odds ratio, *OR* odds Ratio, *SDH* subdural hematoma, *TBI* traumatic brain injury

*Statistical uncertainty for outcome indicators (the less complications, the better the quality of care) was determined by the median number of events/median number of included patients per centre. We set the threshold for poor potential at less than 10 events

^a^Pharmaceutical or mechanical

^b^Based on the IQR

^c^Based on non-significant between-centre differences

### Discriminability

**Fig. 1:**
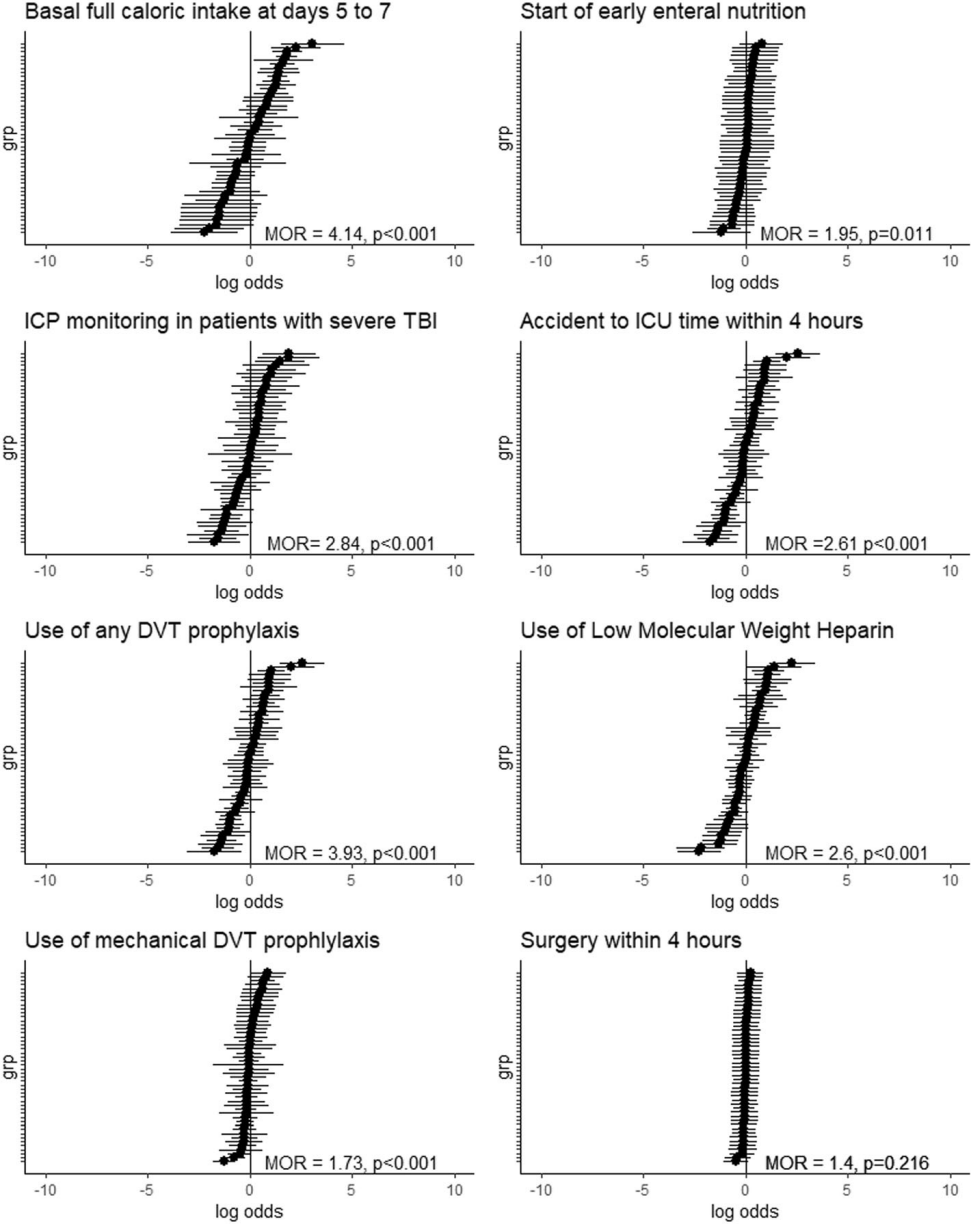
Adjusted random effect estimates per centre for process indicators. This figure shows the between-centre differences for the process indicators (beware of different *x*-axes). Quality indicator definitions can be found in Additional file 3. On the *y*-axis, each dot represents a centre. A centre with an average indicator score has log odds 0 (a positive log odds indicates higher indicator scores and a negative log odds lower indicator scores). The between-centre differences are represented by the shape of the caterpillar plots; the variation in the log odds for individual centres and the corresponding confidence intervals (uncertainty). For example, the use of ICP monitoring shows large variation between centres with small confidence intervals, so there is high variation with low statistical uncertainty. While for use of low molecular weight heparin, the variation is large, but the statistical uncertainty is high as well (due to high adherence rates for most centres). The caterpillars were based on non-missing data (after imputation). ‘Use of Low Molecular Weight Heparin’ reflects the indicator ‘Number of patients that receive pharmaceutical prophylaxis with low molecular weight heparins/ total number of TBI patients admitted to the ICU’. ‘Surgery within 4 hours’ reflects the indicator ‘Median door-to-operation time for acute operation of SDH and EDH with surgical indication’. DVT deep venous thrombosis, EDH epidural hematoma, ICU intensive care unit, MOR median odds ratio, SDH subdural hematoma

Variation in scores between centres was low for structure indicators (with little room for improvement) for ‘existence of a protocol’, ‘availability of a neurosurgeon 24/7 within 30 minutes after call’, and ‘24/7 availability of a CT scan and radiologist review’, due to high overall adherence rates among centres (Additional file 2). For process indicators, high variation was found for all indicators (all MORs above 1.5, all *p* < 0.001) except for ‘surgery within 4 hours in patients with SDH or EDH’ (Fig. 1).

**Fig. 2:**
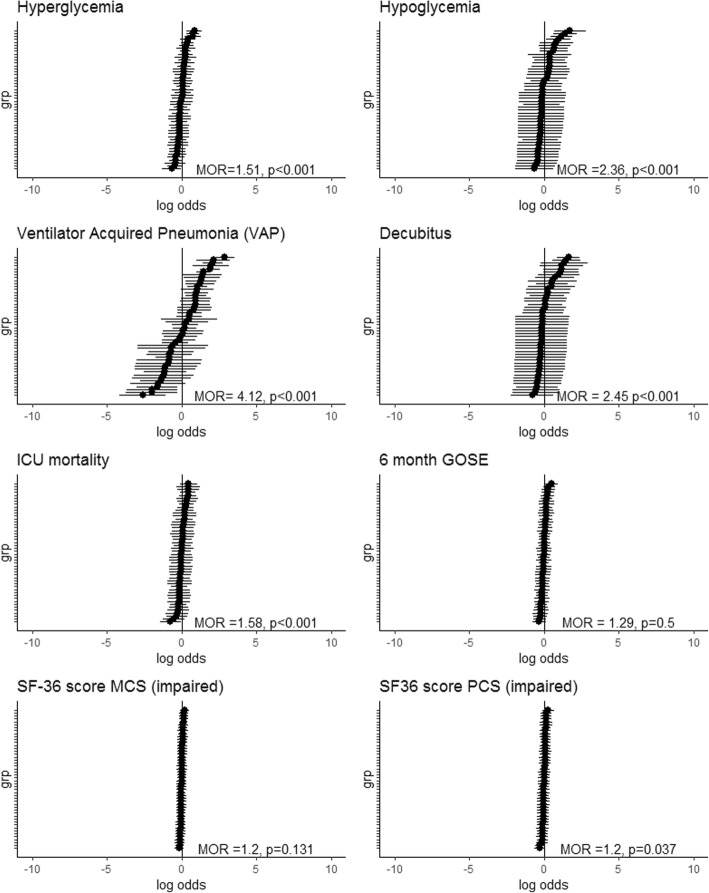
Adjusted random effect estimates per centre for outcome indicators. This figure shows the between-centre differences for the outcome indicators. Quality indicator definitions can be found in Additional file 4. On the *y*-axis, each dot represents a centre. A centre with an average indicator score has log odds 0 (a positive log odds indicates higher indicator scores and a negative log odds a lower indicator scores). Outcome indicator scores were adjusted for case-mix and ‘statistical uncertainty’ (variation by chance) by using a random effects logistic regression model. The MOR (median odds ratio) represents the between-centre variation: the higher the MOR, the larger the between-centre variation (a MOR equal to 1 reflects no variation). The confidence intervals represent the statistical uncertainty. The caterpillars were based on non-missing data (after imputation). Outcome incidence for decubitus and hypoglycemia was too low to reliably show between-centre variation (high confidence intervals). Impaired SF-36v2 (PCS or MCS) score ≤ 40. CI confidence interval, GOSE Glasgow Outcome Scale Extended, ICU intensive care unit, MOR median odds ratio

For outcome indicators, the between-centre variation was significant as well. The variation between centres was especially high for ventilator-acquired pneumonia (VAP) with a MOR of 4.12. Little between-centre variation on the 6-month GOSE was found (MOR = 1.29, *p* = 0.5) (Fig. 2).

Overall, five structure (three with moderate performance), two process, and four outcome indicators showed low discriminability (Table 2).

### Statistical uncertainty

Four indicator definitions were slightly changed without changing its content (Additional files 3 and 4, bold definitions). Median event rates for the outcome indicators hyperglycemia, ICU mortality, and ventilator-associated pneumonia (VAP) were respectively 8, 4, and 3 events per centre. Median event rates for hypoglycemia and decubitus were zero. All these event rates reflect high statistical uncertainty (Additional file 4, Table 2).

## Discussion

We showed that it was feasible to obtain most quality indicators from a recently proposed, consensus-based, quality indicator set for traumatic brain injury (TBI) at the ICU based on sufficient data completeness. The suboptimal adherence scores in combination with between-centre variation suggest a potential for quality improvement, specifically for process and outcome indicators. However, statistical uncertainty was generally high for outcome indicators, making them less suitable for quality improvement purposes and benchmarking in particular. Based on the assessment of feasibility, discriminability, and statistical uncertainty, we found nine structure indicators, five process indicators, but none of the outcome indicator out of 26 indicators to be appropriate for quality measurement and improvement in this validation study. Overall, the quality of ICU care can be improved for patients with TBI, and our analysis provides a useful case of how quality indicators for ICU care in TBI can be evaluated in a large database.

To our knowledge, this is the first quality indicator set to be developed and validated in adult patients with TBI admitted to the ICU. We have summarized quality indicators with the potential to be used for benchmarking and quality improvement. First, we recommend reducing the initial set by excluding indicators with a low percentage available data (low feasibility), in a given dataset. The low feasibility on some process indicators might be explained by the complexity and high resource needs of collecting data on process indicators. However, feasibility could be improved with automatic data extraction in the future. Second, quality indicators with high between-centre variation (most quality indicators in this study) and suboptimal adherence rates (discriminability) can be used to improve quality of care and for benchmarking. Third, event rates of outcome indicators were generally low (even over a study duration of 4 years), indicating that outcome indicators have a low potential for quality improvement in this study population due to high statistical uncertainty. However, the threshold of 10 events might be too strict, or alternatively, outcome indicator denominators should be restricted to patients with a more severe injury, greater organ dysfunction, more interventions, or longer length of stay to increase the number of events and to increase statistical power. Over time, registration and use of the quality indicators could provide further insights into their role in quality improvement and benchmarking and allow their re-evaluation and refinement.

Quality of care in critically ill patients with TBI could potentially be improved in various areas, as indicated by a sub-optimal adherence of European ICUs to most quality indicators. The large (adjusted) between-centre variation suggests that some centres significantly outperform others. Wide sharing of best practice and implementation strategies from centres that perform well on quality indicators describing structures and processes of care and/or registering a low incidence of adverse outcomes could improve performance in centres that perform less well.

Previous studies also report large between-centre differences in processes of TBI care across Europe [24–26]. This between-centre variation could be explained by variation in adherence to guidelines. Although 89% of centres indicated that they complied with the Brain Trauma Foundation (BTF) guidelines, actual assessment of real-time practice may be different. For example, ICP monitoring in patients with severe TBI (GCS < 9) is one of the higher-level evidence recommendations in the BTF guidelines, but we only found adherence rates of 44–82% (IQR) across centres in our study. This implies that there is much to gain in the reduction of variation in evidence-based care processes. One previous study reported the performance of quality indicators in children with TBI [27]. Although their indicators differed from those in the current study, they found a lower variation in adherence rates (between 68% and 78%). Several registries already exist for general ICU [3, 5]—or trauma care [2, 4]. Some of the outcome indicators we tested are also used in current ICU registries but did not perform well in our study (decubitus ulcers and hypoglycemia). For example, in our study, the outcome score for decubitus ulcers approached 0%, while in Dutch hospitals, decubitus was found in around 6% of patients [16].

This study has several strengths. First, we tested the potential of consensus-based quality indicators in a large clinical dataset, while most previous studies only report a Delphi study to develop quality indicators and only a few studies pilot-tested quality indicators before implementation [28, 29]. Second, the indicator scores were derived from the CENTER-TBI database, which includes a substantial number of patients with TBI across many ICUs. Indeed, this analysis provides the first opportunity to study indicator performance and between-centre variation in TBI management on a larger scale. The CENTER-TBI database has only one exclusion criterion, so it represents a cohort generalizable to the TBI population across Europe.

Our study also has some limitations. Staffing and organizational data were only partly captured in CENTER-TBI. The structure indicators were based on questionnaires which might be imprecise. Patients of all severities (including early deaths) were included for analyses. We recognize that a selection of patients with a longer ICU stay may have increased between-centre comparability, but we mitigated this issue by correcting all between-centre analyses for case-mix severity. We defined feasibility as the completeness of the data, while other aspects of feasibility, such as accessibility, timeliness, and missing data at a centre level, could not be addressed [30]. Statistical uncertainty was reflected in the number of event rates, while also other aspects as intra- and inter rater reliability of medical coders are important but could not be addressed. We decided not to test the construct (correlations between indicators) and criterion validity (association with outcome) of the final indicator set as these are hard to test [31]; for construct validity, predetermined correlations between quality indicators are hard to find between different aspects of processes of care and often do not correlate with outcome; and for criterion validity, the case-mix adjustment would differ per quality indicator and even very complex models cannot adjust for all residual bias (unmeasured confounding). However, ongoing evaluation of these quality indicators in larger datasets could include assessment of such correlations with the outcome.

Future implementation of the quality indicators in a European registry will make it possible to monitor TBI patient data over time and among countries. Feedback from this registry to individual ICUs is essential to make stakeholders be aware of their centre performance and help develop internal quality improvement programmes. No reference standards for the quality indicators have been defined. Our study also illustrates some pitfalls, since some of these indicators are quite complex and difficult to assess retrospectively. Such data collection could, however, be optimized by routine registration of timing of events and processes, automatic data extraction, and clear definitions. Overall, the methods illustrated in this study can be used to optimize future data collection (with uniform indicator definitions and data quality), to calculate quality indicators (adjusted across centres) and to identify areas in need of further research (due to high variation).

## Conclusions

This study validated a consensus-base quality indicator set in a large prospective TBI study (CENTER-TBI). Quality of care in critically ill patients with TBI appears amenable to improvement in various areas as indicated by sub-optimal adherence rates and between-centre variation for many quality indicators. Further, our analysis generally shows good feasibility and discriminability but high statistical uncertainty for several outcome indicators. Future research should focus on implementation and quality improvement efforts and continuous reevaluation of the quality indicators.

## Supplementary information

**Additional file 1.** Exclusion of Delphi quality indicators for application to the CENTER-TBI data. This table describes the consensus-based quality indicators (from the Delphi study) that could not be applied to the CENTER-TBI dataset for various reasons.

**Additional file 2.** Structure indicator scores. This table shows the calculated structure indicator scores in the CENTER-TBI study. This is calculated at center-level including missing data and complete cases.

**Additional file 3.** Process indicator scores. This table shows the calculated process indicator scores in the CENTER-TBI study. This is calculated at patient- and center-level including missing data and complete cases.

**Additional file 4.** Outcome indicator scores. This table shows the calculated outcome indicator scores in the CENTER-TBI study. This is calculated at patient- and center-level including missing data and complete cases.

**Additional file 5.** CENTER-TBI investigators and participants for the ICU stratum. This file includes the collaborator group: the CENTER-TBI investigators and participants for the ICU stratum and their affiliations.

## Data Availability

The datasets generated and/or analyzed during the current study are available via https://www.center-tbi.eu/data on reasonable request.

## Abbreviations

BTF: Brain trauma foundation
CENTER-TBI: Collaborative European NeuroTrauma Effectiveness Research in Traumatic Brain Injury
CT: Computed tomography
DVT: Deep venous thrombosis
EDH: Epidural hematoma
GCS: Glasgow Coma Scale
GOSE: Glasgow Outcome Scale Extended
ICP: Intracranial pressure
ICU: Intensive care unit
IMPACT: International Mission for Prognosis and Analysis of Clinical Trials in traumatic brain injury
IQR: Interquartile range
ISS: Injury severity score
MOR: Median odds ratio
SDH: Subdural hematoma
SF-36v2: Short-Form36 version 2
VAP: Ventilator-acquired pneumonia
