# Supplementary material for: Quality indicators for patients with traumatic brain injury in European intensive care units: a CENTER-TBI study
Source: Crit Care. 2020 Mar 4;24:78. doi: 10.1186/s13054-020-2791-0 (PMC7057641; doi:10.1186/s13054-020-2791-0)
Supplement: Supplementary file 1 — Additional file 1. Exclusion of Delphi quality indicators for application to the CENTER-TBI data. This table describes the consensus-based quality indicators (from the Delphi study) that could not be applied to the CENTER-TBI dataset for various reasons. [file 13054_2020_2791_MOESM1_ESM.docx]

| Table 1. Exclusion of Delphi quality indicators for application to the CENTER-TBI data | |
| --- | --- |
| Indicator | Reason for exclusion |
| Structure indicators |  |
| 1. Certified intensivist present in person 7 days a week during at least day-time (yes/no) | 1 |
| 1. Information on prognosis discussed with family by one of the treating physicians (ICU physician or neurosurgical physician) at least once during ICU stay | 2 |
| 1. A daily meeting between intensivist and neurosurgeon to discuss patients with TBI at the ICU (yes/no) | 2 |
| 1. Total number of disciplines (i.e. neurologist, physiotherapy, occupational therapy) involved during ICU stay | 1 |
| 1. 24/7 availability of a certified person at your centre that can insert an ICP monitor within 2 hours after admission at the ICU (yes/no) | 2 |
| 1. The presence of a protocol/ institutional guideline that provide indications for surgery with SDH an EDH (yes/no) | 1 |
| Process indicators | |
| 1. Number of assessments of delirium presence with validated screening tool conscious TBI patients / total number of ICU days in conscious TBI patients | 2 |
| 1. Number of patients with TBI visited daily by a physiotherapist during ICU stay/ total number of patients with TBI at the ICU | 2 |
| 1. Number of patients with TBI receiving follow-up by a specialist within 2 months after discharge/ total number of patients with TBI discharged (not in rehab clinic) | 1 |
| 1. Number of visits by a neurosurgeon/ total number of ICU days in patients with TBI | 2 |
| 1. Number of patients with a support plan (e.g. rehabilitation) after ICU discharge/ number of patients discharged from the ICU | 2 |
| 1. Number of assessments of motor scores of the GCS/ total number of ICU days in patients with TBI | 3 |
| 1. Number of assessments of pupillary responses/ total number of ICU days in patients with TBI | 3 |
| 1. Number of patients with neuropsychological testing at hospital discharge/ number of patients with TBI discharged from the hospital | 3 |
| Outcome indicators | |
| 1. Number of EVD infections in patients with TBI/ total number of patients with TBI at the ICU with an EVD inserted | 1 |
| 1. Number of patients with TBI with severe sepsis or septic shock/ total number of patients with TBI at the ICU | 1 |
| Reasons for exclusion 1) No identical definition between CENTER-TBI database and quality indicator, 2) Underlying variables not reported in study, 3) data collection for the CENTER-TBI study is different from routine data collection in clinical practice, which impacted on the absolute values of the QI (numerator and/or denominator), invalidating the QI result. | |
