# Supplementary material for: Quality indicators for patients with traumatic brain injury in European intensive care units: a CENTER-TBI study
Source: Crit Care. 2020 Mar 4;24:78. doi: 10.1186/s13054-020-2791-0 (PMC7057641; doi:10.1186/s13054-020-2791-0)
Supplement: Supplementary file 2 — Additional file 2. Structure indicator scores. This table shows the calculated structure indicator scores in the CENTER-TBI study. This is calculated at center-level including missing data and complete cases. [file 13054_2020_2791_MOESM2_ESM.docx]

| Table 2. Structure indicator scores | | | |
| --- | --- | --- | --- |
|  | Centre-level (N=54) | | |
| Structure indicators | Number of centres (N) | Missing  (N) | Indicator scores  Complete cases(%) |
| 1. The existence of a protocol including specific guidelines (like the BTF guidelines or institutional guidelines) for Traumatic Brain Injury patients (yes/no) | 47 | 1 | 89% |
| 1. The presence of (some form of) regular audits to check guideline adherence in general at the Intensive Care Unit (ICU) (yes/no)   - Once in the last 5 years  - Annually  - Several times per year  - Overall | 14  1  1  16 | 1 | 26%  2%  2%  30% |
| 1. The presence of dedicated person(s) to oversee guidelines development and maintenance, including those for patients with TBI, at the ICU (yes/no)   - Individual  - Group  - Overall | 11  33  44 | 1 | 21%  62%  83% |
| 1. Does your hospital have a dedicated/specialized neurocritical care unit? (yes/no) | 35 | 0 | 65% |
| 1. The availability of operating rooms 24 hours per day (yes/no) | 40 | 0 | 74% |
| 1. The presence of a step down unit where patients can still be monitored 24/7, but less intensively than at the ICU (yes/no) | 38 | 0 | 70% |
| 1. Intensivist to ICU bed ratio 1 to   - 0-5  - 6-10  - >10 | 27  22  5 | 0 | 50%  41%  9% |
| 1. ICU nurse to ICU bed ratio 1 to   - 0-<1  - 1-2  - >2-3 | 14  23  17 | 0 | 26%  43%  31% |
| 1. Do you have a protocol for glucose management available for patients with TBI at your ICU? yes/no | 43 | 1 | 81% |
| 1. Availability of a neurosurgeon (staff) 24/7 within 30 minutes after call (yes/no) | 49 | 0 | 91% |
| 1. 24/7 availability of a CT scan and radiologist review (yes/no) | 50 | 0 | 93% |
| This table shows the indicator scores of all centres for the structure indicators at both centre and patient-level.  Regarding structure indicators, the missing data is a measure for feasibility and the indicator score for discriminability (the percentage of centres that indicated yes compared with no). For the denominator of the indicator scores the total number of centres (*N* = 54) and admitted patients (*N* = 2138) is taken. The complete cases takes the missing data into account. Structure indicators are extracted from the Provider Prefilling database for those centres that participating in the CENTER-TBI study.  BTF: Brain Trauma Foundation, ICU: Intensive Care Unit, TBI: traumatic brain injury | | | |
