# Supplementary material for: Quality indicators for patients with traumatic brain injury in European intensive care units: a CENTER-TBI study
Source: Crit Care. 2020 Mar 4;24:78. doi: 10.1186/s13054-020-2791-0 (PMC7057641; doi:10.1186/s13054-020-2791-0)
Supplement: Supplementary file 3 — Additional file 3. Process indicator scores. This table shows the calculated process indicator scores in the CENTER-TBI study. This is calculated at patient- and center-level including missing data and complete cases. [file 13054_2020_2791_MOESM3_ESM.docx]

| Table 3. Process indicator scores | | | | | | | |
| --- | --- | --- | --- | --- | --- | --- | --- |
|  | Centre-level (N=54) | | | Patient-level (N=2138) | | | |
|  |  | Complete cases | | Denom |  | Complete cases  Indicator scores | |
|  | Nr of  centres | Median scores | IQR (%) | Nr of patients | Missing |  |  |
| Process indicators | (N) | (%) | (Q1-Q3) | (N) | (%) | Num/denom | (%) |
| 1. Number of TBI patients with basal full caloric replacement within 5 to 7 days post-injury / number of TBI patients ~~at~~ ~~the ICU~~ at day 5 to 7 **post-injury**   Data: 1500/day females or 1750/day males ^a^ | 39 | 20% | 3-47 | 1084 | 0% | 314/1084 | 29% |
| 1. Median accident-to-ICU-admission time (hours)   0-≤4  4-≤8  >8 | 50  50  51 | 35%  41%  21% | 24-50  30-50  10-32 | 2006 | 1% | 820/1980  744/1980  416/1980 | 42%  37%  21% |
| 1. Number of severe (GCS 3-8) TBI patients with ICP monitoring/ number of severe TBI patients at the ICU   Data: baseline GCS ^b^ | 50 | 69% | 44-82 | 915 | 0% | 559/915 | 61% |
| 1. Number of patients with TBI that receive any DVT prophylaxis ^c^/ total number of patients with TBI at the ICU   Any prophylaxis ^c^  Mechanical  Pharmaceutical  Extra: timing within 72 hours | 50  43  50  47 | 80%  40%  65%  38% | 60-94  6-71  54-83  25-81 | 2006 | 3%  5%  3%  36% | 1472/1950  853/1905  1279/1951  639/1274 | 75%  45%  66%  50% |
| 1. Number of patients that receive pharmaceutical prophylaxis with low molecular weight heparins/ total number of TBI patients admitted to the ICU | 47 | 63% | 49-78 | 2006 | 3% | 1244/1948 | 64% |
| 1. Number of patients with TBI that receive mechanical DVT prophylaxis (e.g. stockings) initiated within 6 hours **after ICU admission** / total number of patients with TBI at the ICU with the possibility to receive stockings   Data: initiated within 24 hours | 38 | 71% | 50-91 | 1905 | 57% | 558/821 | 68% |
| 1. Number of TBI patients with start of (early) enteral nutrition within 72 hours **post-injury**/ number of patients with enteral feeding during ICU | 51 | 99% | 87-100 | 1109 | 22% | 806/ 867 | 93% |
| 1. ~~Median~~ door-to-operation time for acute operation of SDH and EDH with surgical indication (hours)   0-≤4  5-≤8  >8  Data: acute operation < 24 hours | 47  33  30 | 64%  15%  18% | 50-79  0-25  0-28 | 377 | 0% | 237/377  60/377  80/377 | 63%  16%  21% |
| This table shows the indicator scores for the process indicators at both centre- and patient-level.  At centre-level, the number of centres represents the number of centres that adhered to the indicator (excluding centres with missing data). For all centres (also non adherent), the median indicator score and IQR is shown (for centres with data available). At patient-level, the indicator scores of complete cases shows the indicator scores taking missing data into account (therefore denominators reflect complete cases). The missing data at patient-level represents the feasibility of an indicator, the IQR and range at centre-level the discriminability. Process indicators are calculated from the CENTER-TBI database. **Bolt** **indicator definitions** were adjusted as felt more appropriate compared with the definition of the Delphi study, based on the actual data available.  a) Available data/ variable from the CENTER-TBI database b) GCS at baseline: Post stabilization value, if absent prehospital values are used. Intubated/ untestable verbal (V) scores are treated as unknown c) Mechanical or pharmaceutical DVT prophylaxis  Denom: denominator (eligible patients), EDH: Epidural hematoma, ICU: Intensive Care Unit, IQR: Interquartile range, Num: numerator, SDH: subdural hematoma, TBI: traumatic brain injury | | | | | | | |
