# Supplementary material for: Quality indicators for patients with traumatic brain injury in European intensive care units: a CENTER-TBI study
Source: Crit Care. 2020 Mar 4;24:78. doi: 10.1186/s13054-020-2791-0 (PMC7057641; doi:10.1186/s13054-020-2791-0)
Supplement: Supplementary file 4 — Additional file 4. Outcome indicator scores. This table shows the calculated outcome indicator scores in the CENTER-TBI study. This is calculated at patient- and center-level including missing data and complete cases. [file 13054_2020_2791_MOESM4_ESM.docx]

| Table 4. Outcome indicator scores | | | | | | | |
| --- | --- | --- | --- | --- | --- | --- | --- |
|  | Centre-level (N=54) | | | Patient-level (N=2138) | | | |
| Outcome indicators | Number of centres  (N) | Complete cases | | Denom  Nr of patients  (N) | Missing  (%) | Complete cases  Indicator scores | |
|  |  | Median scores | IQR  (Q1-Q3) |  |  |  | |
|  |  | (%) | (%) |  |  |  |  |
|  |  |  |  |  |  | Num/denom | (%) |
| 1. Number of TBI patients with any blood glucose above 10 mmol/L (180mg/dL, hyperglycemia)/total number of patients with TBI at the ICU ^a^ | 50 | 35% | 22-45 | 2006 | 7% | 696/1867 | 37% |
| 1. Number of TBI patients with any blood glucose below 4 mmol/L (hypoglycemia)/ total number of patients with TBI at the ICU ^a^ | 21 | 0% | 0-3 | 2006 | 7% | 59/1808 | 3% |
| 1. Number of ICU-deaths among patients with TBI/ total number of ICU-admitted patients with TBI | 46 | 12% | 9-21 | 2006 | 1% | 266/1982 | 13% |
| 1. Incidence of ventilator associated pneumonia ^b^ (VAP) in patients with TBI/ total number of TBI patients with mechanical ventilation at the ICU | 38 | 14% | 0-31 | 1432 | 2% | 249/1410 | 18% |
| 1. Number of TBI patients with decubitus **~~grade 2 or higher~~** at the ICU ^c^/ number of TBI patients at the ICU | 22 | 0% | 0-2 | 2006 | 2% | 45/1961 | 2% |
| 1. The median score of the GOSE from all patients with TBI at 6 months**~~/ number of patients with TBI discharged from the ICU and alive at 6 months~~** | 54 | 5 | 3-7 | 2006 | 14% | 5 | - |
| 1. The median score of the SF-36 from all patients with TBI at 6 months/ number of patients with TBI discharged from the ICU and alive at 6 months   - Physical health ^c^  - Mental health ^d^ | 54  54 | 46  46 | 37-54  36-55 | 1618 | 72% | 45  45 | - |
| This table shows the median percentage or indicator scores (incidence) of the outcome indicators. At centre-level, the number of centres represents the number of centres where the indicator occurs. For all centres (also non adherent), the median indicator score and IQR is shown. At patient-level, for the denominator of the indicator scores the number of patients in the denominator is taken, disregarding the missing data (denominators reflect complete cases).  The missing data at patient-level represents the feasibility of an indicator, the IQR and range at centre-level the discriminability. Outcome indicators are calculated from the CENTER-TBI database. **Bolt** **indicator definitions** were adjusted as felt more appropriate compared with the definition of the Delphi study  a) For available lab results b) No extensive definition available in the data, b) No grade available in the data (only yes/no) c) physical component summary d) Mental component summary  Denom: denominator (all eligible patients), EDH: Epidural hematoma, GOSE: Glasgow Coma Scale Extended, ICU: Intensive Care Unit, IQR: Interquartile range, mg/dL: miligrams per deciliter, mmol/L: millimoles per liter, Num: numerator, SDH: subdural hematoma, SF_36: Short Form (36) Health Survey, TBI: traumatic brain injury | | | | | | | |
