# Supplementary material for: Quality indicators for patients with traumatic brain injury in European intensive care units: a CENTER-TBI study
Source: Crit Care. 2020 Mar 4;24:78. doi: 10.1186/s13054-020-2791-0 (PMC7057641; doi:10.1186/s13054-020-2791-0)
Supplement: Supplementary file 5 — Additional file 5. CENTER-TBI investigators and participants for the ICU stratum. This file includes the collaborator group: the CENTER-TBI investigators and participants for the ICU stratum and their affiliations. [file 13054_2020_2791_MOESM5_ESM.docx]

**The CENTER-TBI ICU WP6 participants and ICU ONLY investigators:**

Cecilia Åkerlund^1^, Krisztina Amrein ^2^, Nada Andelic^3^, Lasse Andreassen^4^, Gérard Audibert^5^, Philippe Azouvi^6^, Maria Luisa Azzolini^7^, Ronald Bartels^8^, Ronny Beer^9^, Bo‑Michael Bellander^10^, Habib Benali^11^, Maurizio Berardino^12^, Luigi Beretta^7^, Erta Beqiri^13^, Morten Blaabjerg^14^, Stine Borgen Lund^15^, Camilla Brorsson^16^, Andras Buki^17^, Manuel Cabeleira^18^, Alessio Caccioppola^19^, Emiliana Calappi^19^, Maria Rosa Calvi^7^, Peter Cameron^20^, Guillermo Carbayo Lozano^21^, Marco Carbonara^19^, Ana M. Castaño‑León^22^, Simona Cavallo^12^, Giorgio Chevallard^13^, Arturo Chieregato^13^, Mark Coburn^24^, Jonathan Coles^25^, Jamie D. Cooper^26^, Marta Correia^27^, Endre Czeiter^17^, Marek Czosnyka^18^, Claire Dahyot‑Fizelier^28^, Paul Dark^29^, Véronique De Keyser^30^, Vincent Degos^11^, Francesco Della Corte^31^, Hugo den Boogert^8^, Bart Depreitere^32^, Dula Dilvesi^33^, Abhishek Dixit^34^, Jens Dreier^35^, Guy‑Loup Dulière^36^, Erzsébet Ezer^37^, Martin Fabricius^38^, Kelly Foks^39^, Shirin Frisvold^40^, Alex Furmanov^41^, Damien Galanaud^11^, Dashiell Gantner^20^, Alexandre Ghuysen^42^, Lelde Giga^43^, Jagos Golubovic^33^, Pedro A. Gomez^22^, Francesca Grossi^31^, Deepak Gupta^44^, Iain Haitsma^45^, Raimund Helbok^9^, Eirik Helseth^46^, Peter J. Hutchinson^47^, Stefan Jankowski^48^, Faye Johnson^49^, Mladen Karan^33^, Angelos G. Kolias^47^, Daniel Kondziella^38^, Evgenios Koraropoulos^34^, Lars‑Owe Koskinen^50^, Noémi Kovács^51^, Ana Kowark^24^, Alfonso Lagares^22^, Steven Laureys^52^, Fiona Lecky^53,54^, Didier Ledoux^52^, Aurelie Lejeune^55^, Roger Lightfoot^56^, Alex Manara^58^, Costanza Martino^59^, Hugues Maréchal^36^, Julia Mattern^60^, Catherine McMahon^61^, Tomas Menovsky^30^, Benoit Misset^52^, Visakh Muraleedharan^62^, Lynnette Murray^20^, Ancuta Negru^63^, David Nelson^1^, Virginia Newcombe^34^, József Nyirádi^2^, Fabrizio Ortolano^19^, Jean‑François Payen^64^, Vincent Perlbarg^11^, Paolo Persona^65^, Wilco Peul^66^, Anna Piippo-Karjalainen^67^, Horia Ples^63^, Inigo Pomposo^21^, Jussi P. Posti^68^, Louis Puybasset^69^, Andreea Radoi^70^, Arminas Ragauskas^71^, Rahul Raj^67^, Jonathan Rhodes^72^, Sophie Richter^34^, Saulius Rocka^71^, Cecilie Roe^73^, Olav Roise^74,75^, Jeffrey V. Rosenfeld^76^, Christina Rosenlund^77^, Guy Rosenthal^41^, Rolf Rossaint^24^, Sandra Rossi^65^, Juan Sahuquillo^70^, Oddrun Sandrød^79^, Oliver Sakowitz^60, 79^, Renan Sanchez‑Porras^79^, Kari Schirmer-Mikalsen^78, 80^, Rico Frederik Schou^81^, Peter Smielewski^18^, Abayomi Sorinola^82^, Emmanuel Stamatakis^34^ Nino Stocchetti^83^, Nina Sundström^84^, Riikka Takala^85^, Viktória Tamás^82^, Tomas Tamosuitis^86^, Olli Tenovuo^68^, Matt Thomas^58^, Dick Tibboel^77^, Christos Tolias^88^, Tony Trapani^19^, Cristina Maria Tudora^63^, Peter Vajkoczy^79^, Shirley Vallance^20^, Egils Valeinis ^43^, Zoltán Vámos^37^, Gregory Van der Steen^30^, Jeroen T.J.M. van Dijck^66^, Thomas A. van Essen^66^, Roel P. J. van Wijk^66^, Alessia Vargiolu^23^, Emmanuel Vega^55^, Anne Vik^80, 90^, Rimantas Vilcinis^86^, Victor Volovici^45^, Daphne Voormolen^57^, Petar Vulekovic^33^, Guy Williams^34^, Stefan Winzeck^34^, Stefan Wolf^91^, Alexander Younsi^60^, Frederick A. Zeiler^34,92^, Agate Ziverte^43^ , Tommaso Zoerle^19^, Hans Clusmann^93^

^1^ Department of Physiology and Pharmacology, Section of Perioperative Medicine and Intensive Care, Karolinska Institutet, Stockholm, Sweden

^2^ János Szentágothai Research Centre, University of Pécs, Pécs, Hungary

^3^ Division of Surgery and Clinical Neuroscience, Department of Physical Medicine and Rehabilitation, Oslo University Hospital and University of Oslo, Oslo, Norway

^4^ Department of Neurosurgery, University Hospital Northern Norway, Tromso, Norway

^5^ Department of Anesthesiology & Intensive Care, University Hospital Nancy, Nancy, France

^6^ Raymond Poincare hospital, Assistance Publique – Hopitaux de Paris, Paris, France

^7^ Department of Anesthesiology & Intensive Care, S Raffaele University Hospital, Milan, Italy

^8^ Department of Neurosurgery, Radboud University Medical Center, Nijmegen, The Netherlands

^9^ Department of Neurology, Neurological Intensive Care Unit, Medical University of Innsbruck, Innsbruck, Austria

^10^ Department of Neurosurgery & Anesthesia & intensive care medicine, Karolinska University Hospital, Stockholm, Sweden

^11^ Anesthesie-Réanimation, Assistance Publique – Hopitaux de Paris, Paris, France

^12^ Department of Anesthesia & ICU, AOU Città della Salute e della Scienza di Torino - Orthopedic and Trauma Center, Torino, Italy

^13^ NeuroIntensive Care, Niguarda Hospital, Milan, Italy

^14^ Department of Neurology, Odense University Hospital, Odense, Denmark

^15^ Department of Public Health and Nursing, Faculty of Medicine and health Sciences, Norwegian University of Science and Technology, NTNU, Trondheim, Norway

**^16^**Department of Surgery and Perioperative Science, **Umeå University, Umeå, Sweden**

^17^ Department of Neurosurgery, Medical School, University of Pécs, Hungary and Neurotrauma Research Group, János Szentágothai Research Centre, University of Pécs, Hungary

^18^ Brain Physics Lab, Division of Neurosurgery, Dept of Clinical Neurosciences, University of Cambridge, Addenbrooke’s Hospital, Cambridge, UK

^19^ Neuro ICU, Fondazione IRCCS Cà Granda Ospedale Maggiore Policlinico, Milan, Italy

^20^ ANZIC Research Centre, Monash University, Department of Epidemiology and Preventive Medicine, Melbourne, Victoria, Australia

^21^ Department of Neurosurgery, Hospital of Cruces, Bilbao, Spain

^22^ Department of Neurosurgery, Hospital Universitario 12 de Octubre, Madrid, Spain

^23^ NeuroIntensive Care, ASST di Monza, Monza, Italy

^24^ Department of Anaesthesiology, University Hospital of Aachen, Aachen, Germany

^25^ Department of Anesthesia & Neurointensive Care, Cambridge University Hospital NHS Foundation Trust, Cambridge, UK

^26^ School of Public Health & PM, Monash University and The Alfred Hospital, Melbourne, Victoria, Australia

^27^ Radiology/MRI department, MRC Cognition and Brain Sciences Unit, Cambridge, UK

^28^ Intensive Care Unit, CHU Poitiers, Potiers, France

^29^ University of Manchester NIHR Biomedical Research Centre, Critical Care Directorate,  Salford Royal Hospital NHS Foundation Trust, Salford, UK.

^30^ Department of Neurosurgery, Antwerp University Hospital and University of Antwerp, Edegem, Belgium

^31^ Department of Anesthesia & Intensive Care, Maggiore Della Carità Hospital, Novara, Italy

^32^ Department of Neurosurgery, University Hospitals Leuven, Leuven, Belgium

^33^ Department of Neurosurgery, Clinical centre of Vojvodina, Faculty of Medicine, University of Novi Sad, Novi Sad, Serbia

^34^ Division of Anaesthesia, University of Cambridge, Addenbrooke’s Hospital, Cambridge, UK

^35^ Center for Stroke Research Berlin, Charité – Universitätsmedizin Berlin, corporate member of Freie Universität Berlin, Humboldt-Universität zu Berlin, and Berlin Institute of Health, Berlin, Germany

^36^ Intensive Care Unit, CHR Citadelle, Liège, Belgium

^37^ Department of Anaesthesiology and Intensive Therapy, University of Pécs, Pécs, Hungary

^38^ Departments of Neurology, Clinical Neurophysiology and Neuroanesthesiology, Region Hovedstaden Rigshospitalet, Copenhagen, Denmark

^39^ Department of Neurology, Erasmus MC, Rotterdam, the Netherlands

^40^ Department of Anesthesiology and Intensive care, University Hospital Northern Norway, Tromso, Norway

^41^ Department of Neurosurgery, Hadassah-hebrew University Medical center, Jerusalem, Israel

^42^ Emergency Department, CHU, Liège, Belgium

^43^ Neurosurgery clinic, Pauls Stradins Clinical University Hospital, Riga, Latvia

^44^ Department of Neurosurgery, Neurosciences Centre & JPN Apex trauma centre, All India Institute of Medical Sciences, New Delhi-110029, India

^45^ Department of Neurosurgery, Erasmus MC, Rotterdam, the Netherlands

^46^ Department of Neurosurgery, Oslo University Hospital, Oslo, Norway

^47^ Division of Neurosurgery, Department of Clinical Neurosciences, Addenbrooke’s Hospital & University of Cambridge, Cambridge, UK

^48^ Neurointensive Care , Sheffield Teaching Hospitals NHS Foundation Trust, Sheffield, UK

^49^ Salford Royal Hospital NHS Foundation Trust Acute Research Delivery Team, Salford, UK

**^50^**Department of Clinical Neuroscience, Neurosurgery, **Umeå University, Umeå, Sweden**

^51^ Hungarian Brain Research Program - Grant No. KTIA_13_NAP-A-II/8, University of Pécs, Pécs, Hungary

^52^ Cyclotron Research Center , University of Liège, Liège, Belgium

^53^ Centre for Urgent and Emergency Care Research (CURE), Health Services Research Section, School of Health and Related Research (ScHARR), University of Sheffield, Sheffield, UK

^54^ Emergency Department, Salford Royal Hospital, Salford UK

^55^ Department of Anesthesiology-Intensive Care, Lille University Hospital, Lille, France

^56^ Department of Anesthesiology & Intensive Care, University Hospitals Southhampton NHS Trust, Southhampton, UK

^57^ Department of Public Health, Erasmus Medical Center-University Medical Center, Rotterdam, The Netherlands

^58^ Intensive Care Unit, Southmead Hospital, Bristol, Bristol, UK

^59^ Department of Anesthesia & Intensive Care,M. Bufalini Hospital, Cesena, Italy

^60^ Department of Neurosurgery, University Hospital Heidelberg, Heidelberg, Germany

^61^ Department of Neurosurgery, The Walton centre NHS Foundation Trust, Liverpool, UK

^62^ Karolinska Institutet, INCF International Neuroinformatics Coordinating Facility, Stockholm, Sweden

^63^ Department of Neurosurgery, Emergency County Hospital Timisoara , Timisoara, Romania

^64^ Department of Anesthesiology & Intensive Care, University Hospital of Grenoble, Grenoble, France

^65^ Department of Anesthesia & Intensive Care, Azienda Ospedaliera Università di Padova, Padova, Italy

^66^ Dept. of Neurosurgery, Leiden University Medical Center, Leiden, The Netherlands and Dept. of Neurosurgery, Medical Center Haaglanden, The Hague, The Netherlands

^67^ Department of Neurosurgery, Helsinki University Central Hospital

^68^ Division of Clinical Neurosciences, Department of Neurosurgery and Turku Brain Injury Centre, Turku University Hospital and University of Turku, Turku, Finland

^69^ Department of Anesthesiology and Critical Care, Pitié -Salpêtrière Teaching Hospital, Assistance Publique, Hôpitaux de Paris and University Pierre et Marie Curie, Paris, France

^70^ Neurotraumatology and Neurosurgery Research Unit (UNINN), Vall d'Hebron Research Institute, Barcelona, Spain

^71^ Department of Neurosurgery, Kaunas University of technology and Vilnius University, Vilnius, Lithuania

^72^ Department of Anaesthesia, Critical Care & Pain Medicine NHS Lothian & University of Edinburg, Edinburgh, UK

^73^ Department of Physical Medicine and Rehabilitation, Oslo University Hospital/University of Oslo, Oslo, Norway

^74^ Division of Orthopedics, Oslo University Hospital, Oslo, Norway

^75^ Institute of Clinical Medicine, Faculty of Medicine, University of Olso, Oslo, Norway

^76^ National Trauma Research Institute, The Alfred Hospital, Monash University, Melbourne, Victoria, Australia

^77^ Department of Neurosurgery, Odense University Hospital, Odense, Denmark

^78^ Department of Anasthesiology and Intensive Care Medicine, St.Olavs Hospital, Trondheim University Hospital, Trondheim, Norway

^79^ Klinik für Neurochirurgie, Klinikum Ludwigsburg, Ludwigsburg, Germany

^80^ Department of Neuromedicine and Movement Science, Norwegian University of Science and Technology, NTNU, Trondheim, Norway

^81^ Department of Neuroanesthesia and Neurointensive Care, Odense University Hospital, Odense, Denmark

^82^ Department of Neurosurgery, University of Pécs, Pécs, Hungary

^83^ Department of Pathophysiology and Transplantation, Milan University, and Neuroscience ICU, Fondazione IRCCS Cà Granda Ospedale Maggiore Policlinico, Milano, Italy

**^84^**Department of Radiation Sciences, Biomedical Engineering, **Umeå University, Umeå, Sweden**

^85^ Perioperative Services, Intensive Care Medicine and Pain Management, Turku University Hospital and University of Turku, Turku, Finland

^86^ Department of Neurosurgery, Kaunas University of Health Sciences, Kaunas, Lithuania

^87^ Intensive Care and Department of Pediatric Surgery, Erasmus Medical Center, Sophia Children’s Hospital, Rotterdam, The Netherlands

^88^ Department of Neurosurgery, Kings college London, London, UK

^89^ Neurologie, Neurochirurgie und Psychiatrie, Charité – Universitätsmedizin Berlin, Berlin, Germany

^90^ Department of Neurosurgery, St.Olavs Hospital, Trondheim University Hospital, Trondheim, Norway

^91^ Department of Neurosurgery, Charité – Universitätsmedizin Berlin, corporate member of Freie Universität Berlin, Humboldt-Universität zu Berlin, and Berlin Institute of Health, Berlin, Germany

^92^ Section of Neurosurgery, Department of Surgery, Rady Faculty of Health Sciences, University of Manitoba, Winnipeg, MB, Canada

^93^ Department of Neurosurgery, University Hospital of Aachen, Aachen, Germany
